# Supplementary material for: The management of psychiatric emergencies in Africa: A scoping review of restraint and seclusion practices in clinical settings and their impacts
Source: Glob Ment Health (Camb). 2025 Sep 19;12:e99. doi: 10.1017/gmh.2025.10052 (PMC12418229; doi:10.1017/gmh.2025.10052)
Supplement: Chakkera et al. supplementary material [file S2054425125100526sup001.docx]

**Appendix A: Search Strategy**

**Topic: The Use of Restraint and Seclusion to Manage Psychiatric Emergencies in Africa**

**Date: 6/1/24**

**Database: PubMed (MEDLINE)**

| 1 | ((("Africa"[Mesh] OR Africa*[tw] OR angola[tw] OR benin[tw] OR botswana[tw] OR "burkina faso"[tw] OR burundi[tw] OR cameroon[tw] OR cape verde[tw] OR "central african republic"[tw] OR chad[tw] OR comoros[tw] OR congo[tw] OR "cote d'ivoire"[tw] OR "ivory coast"[tw] OR congo[tw] OR zaire[tw] OR Djibouti[tw] OR "equatorial guinea"[tw] OR ethiopia[tw] OR eritrea[tw] OR gabon[tw] OR gambia[tw] OR ghana[tw] OR guinea[tw] OR "guinea-bissau"[tw] OR kenya[tw] OR lesotho[tw] OR liberia[tw] OR madagascar[tw] OR malawi[tw] OR mali[tw] OR mauritania[tw] OR mauritius[tw] OR Mayotte[tw] OR mozambique[tw] OR namibia[tw] OR niger[tw] OR nigeria[tw] OR rwanda[tw] OR sahara[tw] OR "saint Helena"[tw] OR "sao tome"[tw] OR senegal[tw] OR seychelles[tw] OR "sierra leone"[tw] OR somalia[tw] OR "south africa"[tw] OR sudan[tw] OR swaziland[tw] OR togo[tw] OR tanzania[tw] OR uganda[tw] OR zambia[tw] OR zimbabwe[tw] OR Algeria[tw] OR Egypt[tw] OR Libya[tw] OR morocco[tw] OR Tunisia[tw] OR sudan*[tw] or sahara*[tw] or algeria*[tw] or egypt*[tw] or libya*[tw] or morocc*[tw] or tunisia*[tw] or Cairo[tw] or Rabat[tw] or Casablanca[tw] or Tripoli[tw] or Algiers[tw] or Fes[tw] or Marrakesh[tw] or Tunis[tw] or Carthage[tw] or Tangier[tw] or Kairouan[tw] or Essaouira[tw] or Luxor[tw] or Bizerte[tw] or "El Aaiun"[tw] or Sousse[tw] or Oran[tw] or Annaba[tw] or Constantine[tw] or Biskra[tw] or Chefchaoouen[tw] or Skikda[tw] or "Sharm El Sheikh"[tw] or Volubilis[tw] or "El Oued"[tw] or Meknes[tw] or "Hippo Regius"[tw] or Djemila or Sfax or Tataouine or "Port Said"[tw] or "Ait Benhaddou"[tw] or Benghazi[tw] or Juba[tw] or Tamanrasette[tw] or merzouga[tw] or "El Djem"[tw] or oujda[tw] or Matmata[tw] or Ghat[tw] or Tabessa[tw] or Giza[tw] or Marj[tw] or Ifrane[tw] or "M'Hamid El Ghizlane"[tw] or Agadir[tw] or Tetouan[tw] or "Shubra El Kheima"[tw] or Tobruk[tw] or Khartoum[tw] or Nyala[tw] or Kassala[tw] or Ubayyid[tw] or Kosti[tw] or Wad Madani[tw] or Qadarif[tw] or Al-Fashir[tw] or Daein[tw] or Damazin[tw] or Geneina[tw] or Merowe[tw] OR "Crozet Islands"[tw] or "Iles Crozet"[tw] or "Scattered Islands"[tw] or "Iles Eparses"[tw] or "Addis Ababa"[tw] or Asmara[tw] or Anananarivo[tw] or Arusha[tw] or Axum[tw] or "Bahir Dar"[tw] or Berbera[tw] or Bulawayo[tw] or Dese[tw] or Eldoret[tw] or Garissa[tw] or Geita[tw] or Gondar[tw] or "Great Rift Valley"[tw] or Hargeisa[tw] or Hargeysa[tw] or Hola[tw] or Jinja[tw] or Iringa[tw] or Kigoma[tw] or Jimma[tw] or Korogwe[tw] or Nairobi[tw] or "Dar es Salaam"[tw] or Mombasa[tw] or Mogadishu[tw] or Dodomoa[tw] or Bujumbura[tw] or Mbeya[tw] or Lusaka[tw] or Harare[tw] or Kakamega[tw] or Kampala[tw] or Kigali[tw] or "Kire Dawa"[tw] or Kikuyu[tw] or Kisumu[tw] or Kitale[tw] or Kitui[tw] or Lilongwe[tw] or "Lake Victoria"[tw] or "Lake Tanganyika"[tw] or Lamu[tw] or Lodwar[tw] or Lokichogio[tw] or Malindi[tw] or Machakos[tw] or Marka[tw] or Machakos[tw] or Maputo[tw] or Maralal[tw] or "Mek'ele"[tw] or Meru[tw] or Musoma[tw] or Mtwara[tw] or Mumias[tw] or Moshi[tw] or Moroni[tw] or Morogoro[tw] or Mwanza[tw] or Naivasha[tw] or Nanyuki[tw] or Nakuru[tw] or Namanga[tw] or Nyeri[tw] or "Port Louis"[tw] or Puntland*[tw] or Nyahururu[tw] or Kismayo[tw] or Ruiru[tw] or "Rwenzori Mountains"[tw] or Sinyanga[tw] or Songea[tw] or tanga[tw] or Tabora[tw] or webuye[tw] or Zanzibar[tw] OR Lagos[tw] or Accra[tw] or Abidjan[tw] or Dakar[tw] or Abobo[tw] or Abuja[tw] or Freetown[tw] or Ouagadougou[tw] or Conakry[tw] or Lome[tw] or Bamako[tw] or Cotonou[tw] or Kumasi[tw] or Monrovia[tw] or Ibadan[tw] or Kano[tw] or "Port harcourt"[tw] or "Benin City"[tw] or "Porto Novo"[tw] or Niamey[tw] or Yamoussoukro[tw] or Banjul[tw] or Timbuktu[tw] or Djenne[tw] or Abomeyu[tw] or Zaria[tw] or Tamale[tw] or Jos[tw] or "Cape Coast"[tw] or Maidugul[tw] or Aba[tw] or Gao[tw] or Calabar[tw] or Warri[tw] or Maiduguri[tw] or "Bobo Dioulasso"[tw] or Parakou[tw] or Djougou[tw] or Bohicon[tw] or "Sekondi Takoradi"[tw] or Sunyani[tw] or Obuasi[tw] or Teshie[tw] or Tema[tw] or Sikasso[tw] or Kalabankoro[tw] or Nouakchott[tw] or "Dakhlet Nouadhibou"[tw] or Ilorin[tw] or Kaduna[tw] or Enugu[tw] or Ikorodu[tw] or Onitsha[tw] or Bauchi[tw] or Akure[tw] or Abeokuta[tw] or Sokoto[tw] or Bouake[tw] or Makeni[tw] or Kaduan[tw] or Sosgbo[tw] or Osogbo[tw] or Gombe[tw] or Ilesa[tw] or Badagry[tw] or makurdi[tw] or Sagamu[tw] or Iseyin[tw] or obbomosho[tw] or Awka[tw] or "Ado Ekiti"[tw] or Nsukka[tw] or Ikeja[tw] or Katsina[tw] or Okene[tw] or Lafia[tw] or Minna[tw] or "Ondo city"[tw] or Umuahia[tw] or Calabar[tw] or Yola[tw] or Pikine[tw] or Touba[tw] or "Thies Nones"[tw] or Kolak[tw] or Ziguinch[tw] or Principe[tw] or Luanda[tw] or lobito[tw] or kuito[tw] or huambo[tw] or Malanje[tw] or Douala[tw] or Yaounde[tw] or Bamenda[tw] or Garoua[tw] or Bafoussam[tw] or Nganoundere[tw] or Maroua[tw] or Kouosseri[tw] or Kumba[tw] or "N'Djamena"[tw] or Moundou[tw] or Bangui[tw] or Bimbo[tw] or Brazzaville[tw] or "Point Noire"[tw] or Kinshasa[tw] or Lubumbashi[tw] or Leopoldville[tw] or Elizabethville[tw] or "Mbuji Mayi"[tw] or Bakwanga[tw] or Bukavu[tw] or Costermansville[tw] or Kananga[tw] or Luluabourg[tw] or Kisangani[tw] or Stanleyville[tw] or Tshikapa[tw] or Koalwezi[tw] or Likasi[tw] or Jadotville[tw] or Goma[tw] or Kikwit[tw] or Uvira[tw] or Bunia[tw] or Mbandaka[tw] or Coquilhatville[tw] or Matadi[tw] or Butembo[tw] or Kabinda[tw] or "Mwene Ditu"[tw] or Isiro[tw] or Paulis[tw] or Boma[tw] or Kindu[tw] or Bata[tw] or Malabo[tw] or Libreville[tw] or Zulu[tw] or Tsonga[tw] or Xhosa[tw] or Swazi[tw] or Ndebele[tw] or Tswana[tw] or Sotho[tw] or BaLunda[tw] or Mbundu[tw] or Ovimbundu[tw] or Chaga[tw] or Sukuma[tw] or Pretoria[tw] or "Cape Town"[tw] or Johannesburg[tw] or Durban[tw] or "Port Elizabeth"[tw] or Bloemfontein[tw] or Windhoek[tw] or Maseru[tw] or Pietermaritz[tw] or Nespruit[tw] or Soweto[tw] or Polokwane[tw] or Limpopo[tw] or Rustenburg[tw] or Mahikeng[tw] or Oudtshroom[tw] or Stellenbosch[tw] or Paarl[tw] or Gaborone[tw] or Luanda[tw] or Cabinda[tw] or Huambo[tw] or Lubango[tw] or Kuit[tw] or Malanje[tw] or Lobito[tw] or Lilongwe[tw] or Blantyre[tw] or Mzuzu[tw] or Maputo[tw] or Matola[tw] or Beira[tw] or Nampula[tw] or Chimoio[tw] or Nacala[tw] or Quelimane[tw] or Lusaka[tw] or Kitwe[tw] or Ndola[tw] or Kabwe[tw] or "Copperbelt Harare"[tw] or Bulawayo[tw] or Chitungwiza[tw] or Mutare[tw] or Masvingo[tw] or Monashonaland[tw] or Manicaland[tw]) NOT ("African Americans"[Mesh] OR "African Americans"[tw] OR "African American"[tw])) | 787.449 |
| --- | --- | --- |
| 2 | (restrain* or seclu*)) AND (psychiatr* OR mental health OR psychiatry OR behavioral health OR psychosis OR psychotic OR schiz* OR violen* OR agitat*) | 11,204 |
| 3 | #1 AND #2 | 157 |

**Database: Embase (via Elsevier)**

| 1 | (africa* OR 'sub-saharan africa'/exp OR 'sub-saharan africa' OR 'north africa'/exp OR 'north africa' OR 'angola'/exp OR angola OR 'benin'/exp OR benin OR 'botswana'/exp OR botswana OR 'burkina faso'/exp OR 'burkina faso' OR 'burundi'/exp OR burundi OR 'cameroon'/exp OR cameroon OR 'cape verde'/exp OR 'cape verde' OR 'central african republic'/exp OR 'central african republic' OR 'chad'/exp OR chad OR 'comoros'/exp OR comoros OR 'congo'/exp OR congo OR 'cote divoire' OR 'ivory coast'/exp OR 'ivory coast' OR 'zaire'/exp OR zaire OR 'djibouti'/exp OR djibouti OR 'equatorial guinea'/exp OR 'equatorial guinea' OR 'ethiopia'/exp OR ethiopia OR 'eritrea'/exp OR eritrea OR 'gabon'/exp OR gabon OR 'gambia'/exp OR gambia OR 'ghana'/exp OR ghana OR 'guinea'/exp OR guinea OR 'guinea-bissau'/exp OR 'guinea-bissau' OR 'kenya'/exp OR kenya OR 'lesotho'/exp OR lesotho OR 'liberia'/exp OR liberia OR 'madagascar'/exp OR madagascar OR 'malawi'/exp OR malawi OR 'mali'/exp OR mali OR 'mauritania'/exp OR mauritania OR 'mauritius'/exp OR mauritius OR 'mozambique'/exp OR mozambique OR 'namibia'/exp OR namibia OR 'niger'/exp OR niger OR 'nigeria'/exp OR nigeria OR 'rwanda'/exp OR rwanda OR 'senegal'/exp OR senegal OR 'seychelles'/exp OR seychelles OR 'sierra leone'/exp OR 'sierra leone' OR 'somalia'/exp OR somalia OR 'south africa'/exp OR 'south africa' OR 'sudan'/exp OR sudan OR 'swaziland'/exp OR swaziland OR 'togo'/exp OR togo OR 'tanzania'/exp OR tanzania OR 'uganda'/exp OR uganda OR 'zambia'/exp OR zambia OR 'zimbabwe'/exp OR zimbabwe OR 'algeria'/exp OR algeria OR 'egypt'/exp OR egypt OR 'libya'/exp OR libya OR 'morocco'/exp OR morocco OR 'tunisia'/exp OR tunisia OR cairo OR rabat OR casablanca OR tripoli OR algiers OR tunis OR tangier OR marrakesh OR fez OR 'lake victoria' OR 'great rift valley') NOT ('african americans'/exp OR 'african americans' OR 'african american'/exp OR 'african american') | 1.542.568 |
| --- | --- | --- |
| 2 | (restrain* OR seclu*) AND (psychiatr* OR "mental health" OR psychiatry OR "behavioral health" OR psychosis OR psychotic OR schiz* OR violen* OR agitat*) | 9,619 |
| 4 | #1 AND #2 | 202 |

**Database: CINAHL (via Ebscohost)**

| 1 | ((africa* OR "sub-Saharan Africa" OR "North Africa" OR angola OR benin OR botswana OR "burkina faso" OR burundi OR cameroon OR "cape verde" OR "central african republic" OR chad OR comoros OR congo OR "cote d'ivoire" OR "ivory coast" OR zaire OR djibouti OR "equatorial guinea" OR ethiopia OR eritrea OR gabon OR gambia OR ghana OR guinea OR "guinea-bissau" OR kenya OR lesotho OR liberia OR madagascar OR malawi OR mali OR mauritania OR mauritius OR mozambique OR namibia OR niger OR nigeria OR rwanda OR senegal OR seychelles OR "sierra leone" OR somalia OR "south africa" OR sudan OR swaziland OR togo OR tanzania OR uganda OR zambia OR zimbabwe OR algeria OR egypt OR libya OR morocco OR tunisia OR cairo OR rabat OR casablanca OR tripoli OR algiers OR tunis OR tangier OR marrakesh OR fez OR "lake victoria" OR "great rift valley")) NOT ("African Americans" OR "African American") | 2,115,794 |
| --- | --- | --- |
| 2 | (restrain* OR seclu*) AND (psychiatr* OR "mental health" OR psychiatry OR "behavioral health" OR psychosis OR psychotic OR schiz* OR violen* OR agitat*)) | 9,967 |
| 4 | #1 AND #2 | 247 |

**Database: PsycInfo**

| 1 | (Africa OR Africa* OR Angola OR Benin OR Botswana OR "Burkina Faso" OR Burundi OR Cameroon OR "Cape Verde" OR "Central African Republic" OR Chad OR Comoros OR "Cote d'Ivoire" OR "Ivory Coast" OR Congo OR Djibouti OR "Equatorial Guinea" OR Ethiopia OR Eritrea OR Gabon OR Gambia OR Ghana OR Guinea OR "Guinea-Bissau" OR Kenya OR Lesotho OR Liberia OR Madagascar OR Malawi OR Mali OR Mauritania OR Mauritius OR Mayotte OR Mozambique OR Namibia OR Niger OR Nigeria OR Rwanda OR "Saint Helena" OR "Sao Tome" OR Senegal OR Seychelles OR "Sierra Leone" OR Somalia OR "South Africa" OR Sudan OR Swaziland OR Togo OR Tanzania OR Uganda OR Zambia OR Zimbabwe OR Algeria OR Egypt OR Libya OR Morocco OR Tunisia OR Sudan* OR Sahara* OR Algeria* OR Egypt* OR Libya* OR Morocc* OR Tunisia*) NOT ("African American" OR "African Americans") | 2,486 |
| --- | --- | --- |
| 2 | (restrain* OR seclu*) AND (psychiatr* OR "mental health" OR psychiatry OR "behavioral health" OR psychosis OR psychotic OR schiz* OR violen* OR agitat*) | 236 |
| 3 | #1 AND #2 | 2 |

**Database: ProQuest Dissertations and Theses**

| 1 | ((africa* OR "sub-Saharan Africa" OR "North Africa" OR angola OR benin OR botswana OR "burkina faso" OR burundi OR cameroon OR "cape verde" OR "central african republic" OR chad OR comoros OR congo OR "cote d'ivoire" OR "ivory coast" OR zaire OR djibouti OR "equatorial guinea" OR ethiopia OR eritrea OR gabon OR gambia OR ghana OR guinea OR "guinea-bissau" OR kenya OR lesotho OR liberia OR madagascar OR malawi OR mali OR mauritania OR mauritius OR mozambique OR namibia OR niger OR nigeria OR rwanda OR senegal OR seychelles OR "sierra leone" OR somalia OR "south africa" OR sudan OR swaziland OR togo OR tanzania OR uganda OR zambia OR zimbabwe OR algeria OR egypt OR libya OR morocco OR tunisia OR cairo OR rabat OR casablanca OR tripoli OR algiers OR tunis OR tangier OR marrakesh OR fez OR "lake victoria" OR "great rift valley")) NOT ("African Americans" OR "African American") | 5,466 |
| --- | --- | --- |
| 2 | (restrain* OR seclu*) AND (psychiatr* OR "mental health" OR psychiatry OR "behavioral health" OR psychosis OR psychotic OR schiz* OR violen* OR agitat*) | 520 |
| 3 | #1 AND #2 | 44 |
